# Evaluation of Nod-Like Receptor (NLR) Effector Domain Interactions

**DOI:** 10.1371/journal.pone.0004931

**Published:** 2009-04-01

**Authors:** Roland N. Wagner, Martina Proell, Thomas A. Kufer, Robert Schwarzenbacher

**Affiliations:** 1 Department of Molecular Biology, University of Salzburg, Salzburg, Austria; 2 Institute of Medical Microbiology, Immunology and Hygiene, University of Cologne, Cologne, Germany; New York University School of Medicine, United States of America

## Abstract

Members of the Nod-like receptor (NLR) family recognize intracellular pathogens and recruit a variety of effector molecules, including pro-caspases and kinases, which in turn are implicated in cytokine processing and NF-κB activation.

In order to elucidate the intricate network of NLR signaling, which is still fragmentary in molecular terms, we applied comprehensive yeast two-hybrid analysis for unbiased evaluation of physical interactions between NLRs and their adaptors (ASC, CARD8) as well as kinase RIPK2 and inflammatory caspases (C1, C2, C4, C5) under identical conditions. Our results confirmed the interaction of NOD1 and NOD2 with RIPK2, and between NLRP3 and ASC, but most importantly, our studies revealed hitherto unrecognized interactions of NOD2 with members of the NLRP subfamily. We found that NOD2 specifically and directly interacts with NLRP1, NLRP3 and NLRP12. Furthermore, we observed homodimerization of the RIPK2 CARD domains and identified residues in NOD2 critical for interaction with RIPK2.

In conclusion, our work provides further evidence for the complex network of protein-protein interactions underlying NLR function.

## Introduction

Nearly a decade ago, the NLR (nucleotide-binding domain and leucine rich repeat containing) family (recently reviewed in [1]–[3]) of intracellular microbial sensors was introduced with the discovery of NOD1 and its role in NF-κB activation [4]. NLR proteins are proposed to survey the cytoplasm for the presence of microbial invaders and endogenous danger signals [5], [6]. Today, it is widely accepted that NLR proteins are critical to the regulation of the innate immune response and, hence, were progressively appreciated for their critical role in host defense to pathogens.

A total of 22 NLR family members have been identified in humans so far (for members and nomenclature see [7] and http://www.genenames.org/genefamily/nacht.html). Individual NLRs specifically recognize microbial derived non-self products such as peptidoglycan-derived molecules [8], [9], viral dsRNA, bacterial toxins, as well as host-derived danger-molecules like uric acid crystals [10]–[12]. Structurally, NLRs are large multi-domain proteins, that contain N-terminal effector domains for binding downstream signaling molecules, a nucleotide-binding oligomerization domain (NACHT), a winged helix (WH), a superhelical (SH) and a C-terminal leucine rich repeat (LRR) receptor domain [13]. NLR proteins are assigned to particular subgroups according to their respective effector domain (PYD, CARD, BIR, and unclassified).

NLRs prevail in the cytoplasm in a dormant form and are activated through direct or indirect binding of ligands to the LRR-receptor domain. Concomitant conformational changes unlock the NACHT domain leading to oligomerization and the formation of a signaling platform, which is capable of eliciting specific immune responses by the recruitment of specific adaptor molecules as well as effector molecules like inflammatory pro-caspases and kinases [14].

The current model of NLR signaling proposes that the CARD-containing NOD proteins NOD1 and NOD2 interact with the CARD-containing kinase RIPK2 (RIP2/RICK) which further leads to the activation of the NF-κB pathway and MAPK pathways [3]. In contrast, the PYD-containing NLRP proteins (formerly named Nalps) drive caspase-activation by binding to the adaptor protein ASC leading to the processing of pro-inflammatory cytokines [15], [16].

Despite their undisputed importance in host defense, definite biological roles for most NLRs await to be assigned. Comprehensive molecular interaction maps are fragmentary and assessment of NLR signaling networks as a whole, hitherto, is hardly in its early stages. Therefore, the functional and mechanistically details of NLR activation as well as the molecular details of the subsequent initiation of signaling cascades remain elusive. Crucial questions, for instance how these receptors distinguish between self, pathogens, commensal bacteria, and endogenous danger signals, are still open.

To substantiate our understanding of NLR function, this study aimed to decipher the molecular mechanisms of NLR signaling by assessing their protein interaction network in an unbiased, systematic approach. Employing comprehensive yeast two-hybrid analysis, we systematically assessed interactions of the NLR effector domains. Described connections were critically evaluated under identical conditions and we were able to identify novel interactions of NOD2 with distinct members of the NLRP subfamily, namely NLRP1, -3 and -12. Furthermore, we observed homodimerization of the RIPK2 CARD domains and, based on molecular modeling and mutational analysis, identified positions in the CARD1 domain of NOD2 that are essential to mediate interaction with RIPK2.

## Results and Discussion

### NLR effector domain interactions – yeast two-hybrid analysis

To further our understanding of protein interactions engaged in NLR signaling events, we subjected effector domains of 11 distinct NLR proteins (NOD1, NOD2, NLRC4, NLRC5, NLRP1, NLRP2, NLRP3, NLRP7, NLRP10, NLRP11, NLRP12), various downstream signaling partners or effectors (RIPK2, CARD8, ASC, PYDC1), as well as CARD domains of assorted caspases (CASP1, CASP2, CASP4, CASP5, CASP9) to systematic yeast two-hybrid analysis (see Methods). Bait und prey design was based on our previous bioinformatical analyses describing comprehensive sequence and structural homology models of NLR CARD and PYD domains [13].

**Figure 1:**
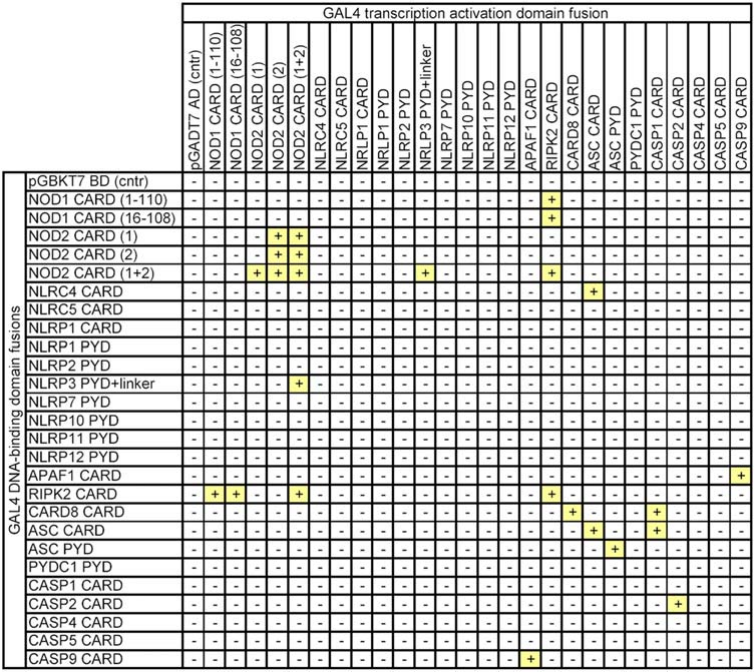
The interaction matrix. In an “each against all” approach an overall number of 676 (26×26) effector domain combinations were analyzed. A “+” indicates an interaction between a particular pair, whereas “−” symbolizes no interaction. In total, the approach yielded 25 distinct associations, which actually corresponded to 7 unique pairs of interacting proteins, as well as 5 homodimerizations.

Preliminary trails revealed feasibility and selectivity of our setup in monitoring NLR effector domain interaction (data not shown). Consequently, we conducted yeast transformations in an “each against all” approach, consisting of an overall number of 676 independent transformations. Thereby, we recorded a total number of 25 interactions (shown in Figure 1), actually corresponding to 12 unique pairs of interacting effector domains (indicated in Table 1). These unique interactions were composed of previously assigned as well as novel associations. Table 1 shows the most important NLR-related protein-protein interactions retrieved from recent scientific literature or selected protein-protein interaction databases (column 1) and indicates whether the respective interactions were observed or not observed in the course of our yeast two-hybrid analysis (column 2). In the following, we will critically contrast our results with recently published data on NLR protein-protein interactions.

**Table 1:** NLR protein-protein interactions.

Interaction	Result	Baits	Preys	Ref.
NOD1 - NLRC4	not observed	NOD1 CARD (1–110), NOD1 CARD (16–108)	NLRC4 CARD	[28]
NOD1 - NLRP1	not observed	NOD1 CARD (1–110), NOD1 CARD (16–108)	NLRP1 PYD, NLRP1 CARD	[59]
NOD1 - RIPK2	observed	**NOD1 CARD (1–110), NOD1 CARD (16–108)**	**RIPK2 CARD**	[4], [60]
NOD1 - CASP1	not observed	NOD1 CARD (1–110), NOD1 CARD (16–108)	CASP1 CARD	[4], [18]
NOD1 - CASP2	not observed	NOD1 CARD (1–110), NOD1 CARD (16–108)	CASP2 CARD	[4]
NOD1 - CASP4	not observed	NOD1 CARD (1–110), NOD1 CARD (16–108)	CASP4 CARD	[4]
NOD1 - CASP9	not observed	NOD1 CARD (1–110), NOD1 CARD (16–108)	CASP9 CARD	[4]
NOD2 - NLRC4	not observed	NOD2 CARD (1), NOD2 CARD (2), NOD2 CARD (1+2)	NLRC4 CARD	[28], [31]
NOD2 - NLRP1	observed§	NOD2 CARD (1), NOD2 CARD (2), **NOD2 CARD (1+2)**	NLRP1 PYD, **NLRP1 PYD+linker**	[29]
NOD2 - NLRP3	observed	NOD2 CARD (1), NOD2 CARD (2), **NOD2 CARD (1+2)**	NLRP3 PYD, **NLRP3 PYD+linker**	[27] *
NOD2 - RIPK2	observed	NOD2 CARD (1), NOD2 CARD (2), **NOD2 CARD (1+2)**	**RIPK2 CARD**	[22]
NOD2 - CASP1	not observed	NOD2 CARD (1), NOD2 CARD (2), NOD2 CARD (1+2)	CASP2 CARD	[28], [29]
NLRC4 - NLRP1	not observed	NLRC4 CARD	NLRP1 PYD, NLRP1 CARD	[28]
NLRC4 - NLRP3	not observed	NLRC4 CARD	NLRP3 PYD+linker	[28]
NLRC4 - NLRP4	not observed	NLRC4 CARD	NLRP4 PYD	[28]
NLRC4 - ASC	observed	**NLRC4 CARD**	**ASC CARD**, ASC PYD	[30], [61]
NLRC4 - CASP1	not observed	NLRC4 CARD	CASP1 CARD	[28], [31], [32]
NLRP1 - APAF1	not observed	NLRP1 CARD, NLRP1 PYD	APAF1 CARD	[59]
NLRP1 - ASC	not observed	NLRP1 CARD, NLRP1 PYD	ASC CARD, ASC PYD, ASC full length	[42]
NLRP1 - CASP1	not observed	NLRP1 CARD, NLRP1 PYD	CASP1 CARD	[33]
NLRP1 - CASP2	not observed	NLRP1 CARD, NLRP1 PYD	CASP2 CARD	[45]
NLRP1 - CASP5	not observed	NLRP1 CARD, NLRP1 PYD	CASP5 CARD	[33]
NLRP1 - CASP9	not observed	NLRP1 CARD, NLRP1 PYD	CASP9 CARD	[45]
NLRP2 - CARD8	not observed	NLRP2 PYD	CARD8 CARD	[40]
NLRP3 - CARD8	not observed	NLRP3 PYD+linker	CARD8 CARD	[40]
NLRP3 - ASC	observed§	NLRP3 PYD+linker, **NLRP3 PYD**	ASC CARD, ASC PYD, **ASC full length**	[43]
NLRP12 - ASC	not observed	NLRP12 PYD	ASC CARD, ASC PYD, ASC full length	[44]
APAF1 - CASP4	not observed	APAF1 CARD	CASP4 CARD	[62]
APAF1 - CASP9	observed	**APAF1 CARD**	**CASP9 CARD**	[63], [64]
RIPK2 - CASP1	not observed	RIPK2 CARD	CASP1 CARD	[41]
CARD8 - CASP1	observed	**CARD8 CARD**	**CASP1 CARD**	[38]
CARD8 - CASP9	not observed	CARD8 CARD	CASP9 CARD	[37]
ASC - CASP1	observed	**ASC CARD**, ASC PYD	**CASP1 CARD**	[33], [34]
ASC - PYDC1	not observed	ASC CARD, ASC PYD	PYDC1 PYD	[35], [36]
**Homodimer/-oligomers**				
NOD1 - NOD1	not observed	NOD1 CARD (1–110), NOD1 CARD (8–108)	NOD1 CARD (1–110), NOD1 CARD (8–108)	[4], [21]
NOD2 - NOD2	observed	**NOD2 CARD (1+2)**	**NOD2 CARD (1+2)**	[22], [23]
NLRP1 - NLRP1	not observed	NLRP1 CARD, NLRP1 PYD	NLRP1 CARD, NLRP1 PYD	[45]
RIPK2 - RIPK2	observed	**RIPK2 CARD**	**RIPK2 CARD**	[17]
CARD8 - CARD8	observed	**CARD8 CARD**	**CARD8 CARD**	[37], [39]
ASC - ASC	observed	**ASC CARD-CARD, ASC PYD-PYD**	**ASC CARD-CARD, ASC PYD-PYD**	[24], [25]
CASP1 - CASP1	not observed	CASP1 CARD	CASP1 CARD	[18]
CASP2 - CASP2	observed	**CASP2 CARD**	**CASP2 CARD**	[26]

Respective protein-protein interactions were mined from the scientific literature or retrieved from MiMI [65]. Results represent data from our yeast two-hybrid analysis (interaction observed or not observed). Baits and preys indicate individual domains tested in our analysis with interacting constructs in bold. References to reported interactions are specified.

§ Initially not observed by “each against all” approach, but observed subsequently with refined constructs.

* No data on a direct interaction.

#### NOD1

Since its first characterization, the binding of NOD1 and RIPK2 was considered a paradigm interaction of NLR signaling and subsequently constituted the foundation of the “induced proximity” model of NF-κB activation by NOD1/RIPK2 [4], [17]. The initial study also described co-immunoprecipitation of NOD1 not only with RIPK2 but also with several pro-caspases, including pro-caspase-1, -2, -4, and -9 [4]. Subsequently, these findings were supported by another report of NOD1-mediated enhancement of IL-1β secretion by direct interaction with pro-caspase-1 [18]. Even though we were able to detect the CARD-CARD-mediated interaction of NOD1 to RIPK2, our two-hybrid data provided no evidence of any direct association of NOD1 CARD with the CARDs of caspase-1, -2, -4, -5, and -9. This is even more intriguing as the caspase-1 and -9 constructs were functional, as assessed by interaction with ASC and Apaf-1, respectively (discussed below).

Recently, NMR as well as crystallographic data on the three-dimensional structure of the NOD1 CARD were presented [19]–[21]. One study suggests CARD homodimerization in a pH-dependent manner [21]. Interestingly, we did not detect any CARD-CARD mediated NOD1 homodimer formation in yeast. However, we observed homodimerization of bacterially overexpressed and purified NOD1 CARDs in solution (data not shown). These discrepancies of *in vivo* and *in vitro* data presumably reflect the requirement of considerably elevated local NOD1 CARD concentrations and challenge the relevance of CARD-mediated homodimer formation *in vivo*, in particular as endogenous NOD1 levels are thought to be very low.

On the other hand, we obtained two-hybrid data for other effector domains reinforcing the significance of CARD-CARD-mediated homodimerization of NLR family members. In accordance with recent reports, we observed NOD2 dimerization in a CARD-dependent manner [22], [23]. Furthermore, we detected an assortment of other CARD-CARD homodimers of non-NLR proteins, including the previously described homodimerizations of ASC [24], [25], and caspase-2 [26], as well as the hitherto neglected intrinsic tendency of RIPK2 CARDs to form homodimers, which is also reflected by its tendency to form aggregates in living human cells (data not shown). These interactions were highly specific for the indicated proteins since homodimers were not detected for any other CARD-containing proteins.

#### NOD2

In addition to the NOD1/RIPK2 interaction we were able to observe the interaction of NOD2 and RIPK2 [22]. Interestingly, on their own neither CARD1, nor CARD2 of NOD2 was sufficient for the interaction. A RIPK2 interaction could only be observed with a construct comprising both N-terminal CARD domains of NOD2. These data agree with the primary literature and are supported by a recent study describing a naturally occurring short variant of NOD2 (NOD2-S) featuring the first and part of the second CARD of the full length protein, which maintains the interaction to RIP2K [23].

Furthermore, we revealed a hitherto unknown NOD2 interaction with NLRP3. Similar to the interaction of NOD2/RIPK2, we observed NLRP3 binding to be dependent on both NOD2 CARD domains. This finding is supported by a recent study, which postulates a functional link between NOD2 and the NLRP3 inflammasome based on the requirement of both NOD2 and NLRP3 for MDP mediated IL-1β release [27]. This interaction was speculated to be dependent on a direct CARD-independent interaction of these proteins.

Although a direct interaction of NOD2 and caspase-1 was previously reported [28], [29] we did not observe a direct interaction of NOD2 as well as NOD1 with caspases-1, -2, -4, -5, and -9 in our system. Taken together this suggests that the NLRC proteins impact on IL-1β activation rather by influencing the NLRP inflammasomes than by direct interaction with caspase-1.

#### NLRC4 (IPAF)

In line with the literature, we found a CARD-CARD-mediated interaction of NLRC4 and ASC [30]. However, we did not observe a direct interaction with caspase-1 [30]–[32]. Furthermore, we found no evidence of an interaction with other NLR proteins, including NOD1, NOD2, NLRP1, NLRP3, and NLRP4 [28], [31]. While the interaction of NLRC4 and caspase-1 reportedly is CARD-CARD-mediated, NLRC4 is supposed to associate with other NLR family members through heterotypic NACHT-domain interactions [28].

#### Adaptor proteins

We found oligomerization of ASC (CARD5, PYCARD) to be mediated by both the CARD as well as the pyrin domain. The underlying pattern of interactions during ASC oligomerization was described in detail previously [25]. Our two-hybrid data foster the importance of both CARD and PYD during the ASC oligomerization process. We were, however, not able to detect a postulated CARD-PYD heterotypic association, indicating that ASC oligomer formation predominantly proceeds via homotypic CARD-CARD as well as PYD-PYD interactions.

In addition, we confirmed a CARD-CARD-based interaction of ASC and caspase-1, which was described previously [33], [34]. Conversely, no interaction of ASC and its prospective inhibitor PYDC1 (POP1) was observed [35], [36].

For CARD8 (CARDINAL, TUCAN), another prospective component of the inflammasome, we confirmed formation of homodimers and interaction with caspase-1 [37]–[39], whereas neither binding to NLRP2 nor NLRP3 was observed [40].

Finally, no direct interactions of RIPK2 with the CARD of caspase-1 [41] could be detected.

#### NLPRs (NALPs)

To date, downstream signal transducers for the majority of NLRP proteins remain largely unknown. As one of only a few examples ASC was demonstrated to mediate downstream NLRP signaling via direct interaction with NLRP1 [42], NLRP3 [43], and NLRP12 [44] in biochemical assays. Furthermore, a direct binding of NLRP1 to caspase-1 and caspase-5 [33], as well as caspase-2 and caspase-9 [45] was proposed.

Remarkably, we could not detect a homotypic PYD-PYD interaction between the NLRP proteins (NLRP1, NLRP2, NLRP3, NLRP7, NLRP10, NLRP11, and NLRP12) and the adaptor protein ASC in our initial “each against all” approach. The experiment was carried out with ASC-PYD (residues 1–94). Since interaction of ASC and NLRP3 is supported by distinct evidence, including genetic interaction data (reviewed in [15]) we refined our constructs. Indeed we observed interaction of ASC full length (residues 1–195) with NLRP3 PYD (residues 1–101). Nonetheless, a direct binding of ASC to other NLRP proteins, including NLRP1 and NLRP12, was still not observed.

Consequently, these findings led us to postulate the presence of a new adaptor or effector protein that serves in connecting NLRP family members to downstream signaling pathways.

#### Strength of interactions

**Figure 2:**
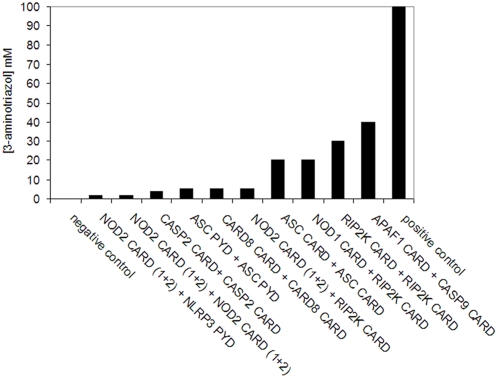
Strength of selected interactions in terms of HIS3 reporter gene activation. The maximum concentration of 3-aminotrizol supporting visible growth of transformants is indicated.

Currently the strength of NLR effector-domain interactions was not evaluated in detail. To this end, we titrated the strength of the detected interactions with 3-aminotriazol (3-AT). This revealed considerable variation in the binding affinities of particular pairs of interacting proteins (Figure 2). The APAF-1 caspase-9 interaction scored strongest in terms of HIS3 reporter gene activation, followed by RIPK2 CARD homodimer formation. The CARD-CARD-dependent interaction of NOD1 and RIPK2 as well as the homodimerization of the ASC CARD domains were comparable in strength, although lower then the ones reported before. In relation, the formation of ASC PYD-PYD homodimers as well the CARD-mediated homodimerizations of caspase-2, CARD8, and NOD2 appeared substantially weak. Interestingly, NOD2 displayed a significantly lower affinity for RIPK2 compared to NOD1. Furthermore, NOD2 binding to NLRP3 apparently is of transient nature as indicated by only slight activation of the reporter genes.

### Interactions in detail

#### RIPK2 homodimerization/-oligomerization

**Figure 3:**
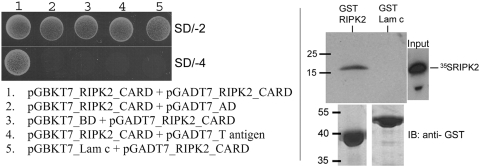
RIPK2 CARD forms homodimers/-oligomers. *Left panel*, Yeast two-hybrid analysis revealed that RIPK2 CARD (residues 427–527) forms homodimers/-oligomers (column 1 on SD/-4). Lamin c (Lam c) was used as control (column 5). *Right panel*, GST pull down assay. Specific binding of ^35^S-labeled RIPK2 CARD (residues 427–527) was observed to recombinant expressed GST-RIPK2 CARD (residues 427–527), whereas binding to GST-Lam c was not detected. SD/-2: SD/-Leu/-Trp, SD/-4: SD/-Ade/-His/-Leu/-Trp.

Figure 3 illustrates the homotypic interaction of RIPK2 CARDs. Two-hybrid data (left panel) were confirmed in vitro by GST pull down analysis (right panel). The interaction proved to be specific since no binding to unrelated proteins was observed.

Signal transduction downstream of NOD1 and NOD2 largely depends on RIPK2. Upon activation, NOD1 and NOD2 recruit RIPK2 via homotypic CARD-CARD interactions [17], [22]. The model of “induced proximity” postulates that enforced oligomer formation of RIPK2 subsequently leads to NOD-dependent activation of NF-κB. Our data add an additional layer of complexity in so far as they reveal a NOD-independent RIPK2 CARD dimer/-oligomer formation. Interestingly, *in vitro* RIPK2 CARD displays strong disposition towards formation of oligomers when subjected to size exclusion chromatography and forms aggregates when expressed in human cells (data not shown). Since we analyzed the mere CARD of RIPK2, further experiments will be needed to determine whether full length RIPK2 actually forms dimers or oligomers *in vivo* and how this relates to control activation of NF-κB downstream of NOD1/2. However, the strong tendency of ASC towards formation of aggregates in the course of apoptosis as well as ASC oligomerization during apoptosis and inflammation are well characterized [24], [38], [46], [47]. Recently, it was shown that caspase-1 is activated during a particular form of apoptosis induced by bacteria, termed pyroptosis, by a large supramolecular complex termed the pyroptosome that is mostly composed of dimers of ASC [47]. It is exciting, that ASC and RIPK2, the essential and best studied downstream adaptor molecules of NLR signaling, display a strong disposition concerning the formation of homodimers/-oligomers. Whether RIPK2 aggregation is of functional importance *in vivo* remains to be determined.

#### NOD2/RIPK2 interaction - analysis of critical residues

Mutations in NOD2 are associated with susceptibility to granulomatous disorders like Blau syndrome and Crohn's disease [48]–[51]. Recently, systematic mutational analysis revealed various residues within the CARDs of NOD2 to be critical for RIPK2-mediated NF-κB activation [52]. It was shown that mutation of single residues can disrupt the interaction of NOD2 and RIPK2 and to abrogate NF-κB activation in response to MDP.

**Figure 4:**
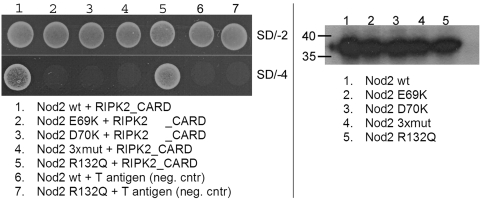
Interaction of distinct NOD2 mutants with RIPK2. *Left panel*, Yeast two-hybrid analysis. NOD2 CARD1+2 interacts with RIPK2 CARD (column 1). No interactions were observed for NOD2 variants harboring a disrupted acidic patch (transformation 2, 3, and 4). An unrelated mutation not affecting the interaction interface had no effect (column 5). *Right panel*, Autoradiography of an *in vitro* transcription/translation of NOD2 proteins. NOD2 constructs were expressed using similar amounts of DNA and lysates.

Our previous bioinformatic analysis of NLR effector domains revealed a high conservation of residues in the acidic patch of the first NOD2 CARD [13]. Accordingly, we generated two different single amino acid substitutions (E69K, D70K), as well as a triple mutation (combining E69K, D70K and E71K) within this acidic surface. These mutants were monitored for RIPK2 binding. We observed that the introduction of single amino acid substitutions within the acidic patch of the CARD1 domain is sufficient to abolish binding to RIPK2 (Figure 4, *left panel*). Neither the E69K nor the D70K substitution of the CARD1-domain interacted with RIPK2. Consistently, also a triple mutation failed to associate with RIPK2, whereas an unrelated mutation (R132Q) outside the anticipated interaction interface did not detectably affect binding affinity. Impaired RIPK2 binding of mutant proteins was not merely due to protein instability as demonstrated by similar expression levels (Figure 4, *right panel*).

Recently, the structure of the NOD1 CARD domain was solved both by NMR and X-ray diffraction [19]–[21]. Based on the published structure Manon et al. identified residues involved in the CARD-CARD interaction of RIPK2 and NOD1 [20]. They were able to demonstrate that the interaction is critically dependent on three acidic residues in NOD1 CARD and three basic residues in RIPK2 CARD. These residues of NOD1 correspond to the residues in NOD2 we found to be crucial for RIPK2 interaction. Thus, it is likely that both CARD-CARD interactions have a strong electrostatic component, similar to the characterized CARD-CARD interaction of APAF1 and caspase-9.

In conclusion, our data confirm the predicted interaction interface and reveal the mode of NOD2/RIPK2 interaction to be similar to the binding of APAF1 and caspase-9.

#### NOD2 directly interacts with NLRP1, NLRP3, and NLRP12

During initial screening for NLR effector domain interactions, we obtained data in support of a direct NOD2/NLRP3 interaction (Figure 1). We found both CARD domains of NOD2 to be essential for this interaction (data not shown). On their own, neither the CARD1, nor the CARD2 domain of NOD2 could mediate significant binding to NLRP3. Initial screening showed that a longer fragment of NLRP3 (residues 1–189) spanning the PYD-domain and a linker-region region proximal to the NACHT domain of NLRP3 did interact with the NOD2 CARD domains. However, we observed that the PYD domain (residues 1–101) of NLRP3 was not sufficient to mediate this interaction. This suggested that the linker region (residues 101–189) between the PYD domain and NACHT domain of NLRP3 comprises the binding surface for NOD2. Indeed we were able to show in subsequent experiments that this particular region of NLRPs is necessary and sufficient for interaction with NOD2 (see below).

Consequently, we re-assessed NLRP constructs (NLRP1, 2, 7, 10, 11, 12) containing this linker region for putative interactions with NOD2 (see Methods, Table 2).

**Table 2:** Cloning of constructs.

Construct name	Accession	Residue range
***Initial “screening”***		
NOD1 CARD (1–110)	AF126484	1–110
NOD1 CARD (16–108)	AF126484	16–108
NOD2 CARD (1)	AF178930	1–116
NOD2 CARD (2)	AF178930	135–255
NOD2 CARD (1+2)	AF178930	1–267
NLRC4 CARD	AF376061	1–96
NLRC5 CARD	AF389420	1–98
NLRP1 CARD	AB023143	1373–1465
NLRP1 PYD	AB023143	1–91
NLRP2 PYD	AK000517	1–95
NLRP3 PYD+linker	AF054176	1–189
NLRP7 PYD	AF464765	1–93
NLRP10 PYD	AY154465	1–96
NLRP11 PYD	AY095145	1–94
NLRP12 PYD	AY095146	1–98
APAF1 CARD	AF013263	1–92
RIPK2 CARD	AF027706	427–527
CARD8 CARD	AF322184	342–430
ASC CARD	AB023416	105–195
ASC PYD	AB023416	1–94
PYDC1 PYD	AF454669	1–95
CASP1 CARD	X65019	1–91
CASP2 CARD	BC002427	32–121
CASP4 CARD	Z48810	1–89
CASP5 CARD	U28015	1–128
CASP9 CARD	U60521	1–97
***Interactions in detail***		
NOD2 CARD (1+2) E69K	AF178930	1–267
NOD2 CARD (1+2) D70K	AF178930	1–267
NOD2 CARD (1+2) 3xmut*	AF178930	1–267
NOD2 CARD (1+2) R132Q	AF178930	1–267
NOD2-S	AF178930	1–180
NOD2 1–163	AF178930	1–163
NOD2 1–135	AF178930	1–135
NOD2 117–267	AF178930	117–267
NLRP1, PYD+linker	AF298548	1–341
NLRP1 linker	AF298548	92–341
NLRP2, PYD+linker	AK000517	1–220
NLRP3, PYD	AF054176	1–101
NLRP7, PYD+linker	AF464765	1–185
NLRP10, PYD+linker	AY154465	1–180
NLRP11, PYD+linker	AY095145	1–160
NLRP12, PYD+linker	AY095146	1–224
ASC full length	AB023416	1–195

GenBank [66] accession numbers, featured domains as well as corresponding amino acid residues are indicated, respectively.

* NOD2 tripled mutation – E69K, D70K, D71K; CARD: caspase activation and recruitment domain, PYD: pyrin domain.

**Figure 5:**
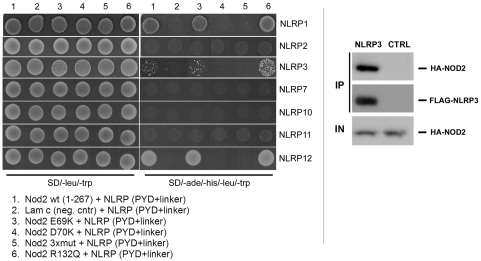
NOD2 directly interacts with NLRP1, NLRP3, and NLRP12. *Left panel*, Yeast two-hybrid analysis of NOD2 CARDs (residues 1–267) showed interaction with NLRP1,-3 and -12, but not with other NLRP proteins (NLRP2, -7, -10, or -11; column 1). Lam c was used as a negative control (column 2). Interestingly, NOD2 mutant E69K maintained the binding to NLRP1, -3, and -12 (column 3), whereas NOD2 D70K (column 4) as well as the NOD2 triple mutant (3xmut, column 5) did not. An unrelated mutation not located within the prospective interaction interface had no effect (column 6). *Right panel*, Physical interaction of NOD2 and NLRP3 in human cells. Western analysis of lysates (IN) and immunoprecipitated complexes (IP) from HEK293T cells, transiently transfected with expression plasmid encoding human HA-NOD2 and FLAG-NLRP3. NLRP3 was immunoprecipitated from cell lysates using a FLAG-epitope specific antibody. Proteins were detected using anti-HA and anti-FLAG antibodies, respectively. As negative control, proteins were immunoprecipitated with FLAG-epitope specific antibody from lysates of HEK293T cells transiently transfected with HA-NOD2, but not FLAG-NLRP3.

Indeed, we now obtained direct interaction of NOD2 and NLRP1 in a CARD-dependent manner (Figure 5A). Additionally, we found an interaction between NOD2 and NLRP12. Taken together, NOD2 CARD1+2 did interact with at least three different NLRP proteins, namely NLRP1, NLRP3, and NLRP12 however did not associate with NLRP2, NLRP7, NLRP10 and NLRP11.

In order to substantiate our two-hybrid data, we performed *in vivo* co-immunoprecipitation experiments (Figure 5B). These experiments showed a specific physical interaction of NOD2 and NLRP3 also in human HEK293T cells.

Interestingly, our data revealed remarkable similarities of NOD2 binding to RIPK2 and NLRP members. In particular, neither the first, nor the second CARD of NOD2 was sufficient for the interaction. Importantly, certain mutations (D70K, triple mutation) within the acidic patch of NOD2 abolished the interaction. The E69K mutation, however, abrogated NOD2 association with RIPK2, but not NLRP1, 3, or 12. Consistently, an unrelated mutation remote to the acidic patch (R132Q) had no effect. These results are consistent with our finding that the NOD2/RIPK2 interaction is CARD-CARD-mediated, while the NOD2/NLRP association employs an as yet to be defined interaction motif located within the NLRP linker region. However, it is likely that the NOD2/NLRP interaction also has a strong electrostatic component, similar to characterized CARD-CARD interactions. Hence, we propose the occurrence of a basic patch within the NLRP linker region. It will be interesting to determine, whether this basic patch is shared by all NLRP members that show an interaction with NOD2.

#### NOD2-S interacts with a linker region of NLRP1

An alternatively spliced, short isoform of NOD2 was recently identified in human cells [23]. NOD2-S comprises the first 180 residues of NOD2 and was shown to interfere with NOD2-induced NF-κB activation and IL-1β release in overexpression assays. It was suggested that this effect is mediated by a direct interaction with both, NOD2 and RIPK2. In this vein, NOD2-S is supposed to inhibit NOD2 oligomerization.

Given the similarities of the NOD2/RIPK2 and the NOD2/NLRP interaction described above, and in light of the affect of NOD2-S on IL-1β release, which may be functionally mediated by NLRPs [27], [29], we monitored distinct NLRP constructs for interaction with NOD2-S (residues 1–180).

**Figure 6:**
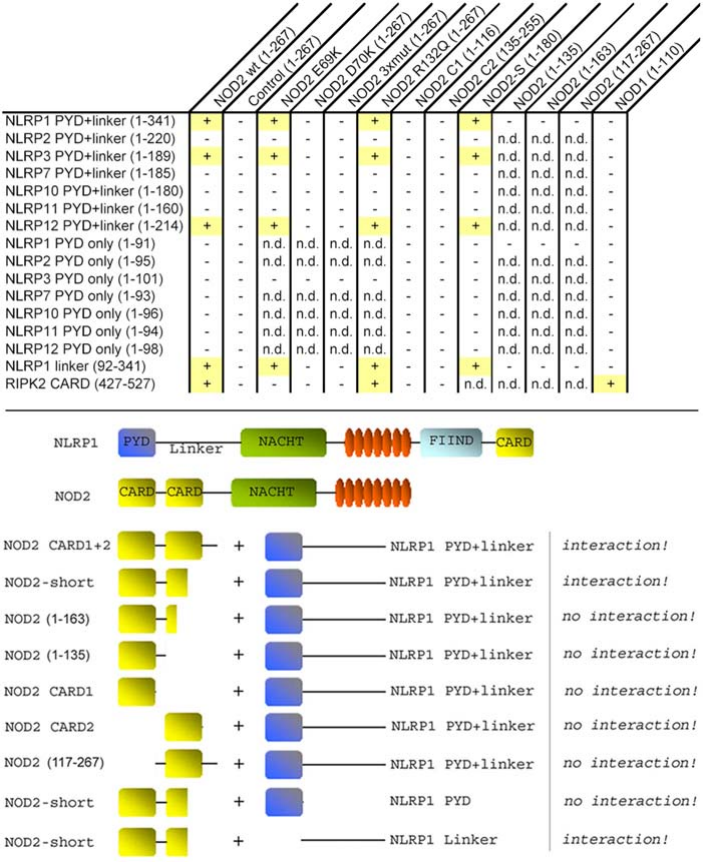
NOD2 interaction with distinct NLRP members. *Upper panel*, A “+” indicates an interaction, “−” symbolizes no interaction (n.d.: not done). A short isoform of NOD2 (NOD2-S, residues 1–180), maintains the interaction with NLRP1, -3, and -12. Furthermore, a linker region within NLRP1 (residues 92–341) proved sufficient for interaction with NOD2. *Lower panel*, Schematic illustration of particular NOD2/NLRP1 constructs and their respective interactions. FIIND: Function to find domain.

In line with the results obtained with the NOD2 CARD domains, NOD2-S interacted with NLRP1, NLRP3, and NLRP12, but not with other NLRP members (Figure 6).

Since, neither the first nor the second CARD domain of NOD2 was sufficient for the interaction, we hypothesized that a region connecting CARD1 and CARD2 might play an essential role in NLRP binding.

To address whether actually this region between the two CARDs of NOD2 is sufficient for NLRP interaction, we analyzed three additional constructs (see Table 2). Surprisingly, none of these NOD2 constructs proved to be capable of interaction with NLRP1 (Figure 6). Therefore, NOD2-S seems to represent the shortest version of NOD2 with NLRP1 binding capacity, since further C-terminal truncations, apparently abrogate the binding, as exemplified by the absence of a detectable NLRP1 interaction for NOD2 1–163 and NOD2 1–135. Moreover, a NOD2 construct encompassing a region immediately succeeding the CARD1 (residues 117–267) failed to interact with NLRP1. In conjunction with our observation that a single point mutation (D70K) in NOD2 CARD1 is capable of interfering with NLRP binding, we therefore propose that in addition to the acidic patch located in CARD1, the proper overall folding of CARD1 and at least part of the CARD2 are prerequisite for interaction of NOD2 with NRLP proteins as well as RIPK2.

In addition, NLRP constructs embracing only the pyrin domain without the adjacent linker region failed to interact with NOD2-S (Figure 6). Excitingly, our data suggests the PYD to be entirely dispensable for the NOD2 interaction. For NLRP1, indeed we can show that a linker region (residues 92–341) is sufficient for NOD2 interaction (Figure 6). Finally, NLRP1, -3, and -12 specifically interact with NOD2, since no binding to NOD1 (residues 1–110) was observed (Figure 6).

Taken together, our results considerably narrow the prospective range of residues necessary for a stable NOD2/NLRP1 interaction by demonstrating that the constructs NOD2-S (1–180) and NLRP1 linker (92–341) are still capable of an interaction, while further truncated versions of NOD2 are not.

### Concluding remarks

Regardless of the enormous interest in NLR function during innate immune response, detailed interaction maps are still not at hand. To our knowledge, no systematic analysis of NLR protein-protein interactions was conducted so far. Here, we present a comprehensive analysis of NLR effector domain interactions. Our analysis confirmed several well known interactions, like the ones of NOD1/NOD2 and RIPK2, providing a good proof of principle for the method used, however, we observed surprisingly few interactions with adaptor molecules, especially ASC. Even though we found an interaction of ASC and caspase-1, as well as homotypic dimerization of the ASC CARD and PYD domain, we did not observe any interactions with NLRP proteins. Instead, we demonstrated a novel NOD2/NLRP connection. Thus, our data on the one hand further substantiate the complexity of NLR signaling, but on the other hand suggests significant gaps in our understanding of NLR signaling pathways.

Even though the NLRP subfamily comprises 14 distinct members, at present information on direct interaction partners is only available for NLRP1, NLRP3 and NLRP12. The role of ASC in formation of the NLRP3 inflammasome is well established [40], however, its involvement in downstream signaling of other NLRP members is less clear. Interestingly, *in vitro* data rule out a strict role for ASC in activation of the NLRP1 inflammasome [53].

Therefore, we propose the existence of as yet unknown downstream interaction partners for NLRP proteins. Since, NLRPs are supposed to signal via their N-terminal effector domains and concomitant homophilic protein-protein interactions, possible contenders for hitherto unknown interactions are likely to be found within the death domain (DD) superfamily (recently reviewed in [54]. The DD superfamily comprises four subfamilies, including the subfamilies of CARD and PYD-containing proteins. The human genome reveals more than 80 DD superfamily members, implicated primarily in the assembly of apoptotic and inflammatory complexes. Interestingly, some viruses have acquired DD-containing sequences that interfere with host apoptotic and inflammatory responses [55]. It is tempting to speculate that future research will not only uncover human DD proteins but also DD homologues in pathogens as NLRP interaction partners.

However, it was revealed by a recent report that putative NLRP interacting proteins not necessarily have to belong to the DD superfamily. It was found that Fas-associated factor 1 (FAF1), a negative regulator of an NF-κB signaling, directly interacts with the PYD domains of NLRP2, NRLP3, NRLP7, NRLP10, and NLRP12 [56].

Yet another possible explanation for the few observed NLRP downstream interaction partners, may be provided by recently observed heterotypic interactions among NACHT-domains of NLRC4 and other NLR proteins (NOD1, NOD2, NLRP1) [28]. In this vein, certain NLRP members (e.g. NLRP10) may exert their function as negative regulators of other NLRP proteins (e.g. NLRRP3) by NACHT-NACHT interactions.

The present study revealed further evidence for the complex interconnections of NLR proteins, however, based on interactions not involving the NACHT-domains. We found NOD2 to interact with NLRP1, NLRP3, and NLRP12. These results suggest an intimate connection or a complex crosstalk between NOD2 and NLRP signaling.

Recently, data on a prospective connection between NOD2 and NLRP signaling pathways began to emerge. It was shown that MDP-induced IL-1β release requires both NOD2 as well as NLRP3 [27]. The group assigned essential, non-redundant roles for NOD2 and NLRP3 in processing pro-IL-1β, and proposed NOD2 to be part of the NLRP3 inflammasome on the basis of a CARD-independent interaction. However, the group referred to the requirement of further studies to determine whether NOD2 and NRLP3 are indeed part of the same complex. The data presented in our study reveal a direct interaction of NLRP3 and NOD2, in a CARD-dependent manner. We therefore anticipate that both proteins are in fact part of the inflammasome. In particular as the interaction surface for NOD2 does not comprise the PYD of NLRP3, leaving the possibility that NLRP3 can still recruit ASC and activate caspase-1 when complexed with NOD2.

Notably, during implementation of our experiments another report on the NOD2/NLRP connection was released [29]. This group reported co-immunoprecipitation of epitope-tagged NOD2 and NLRP1 in transiently transfected HEK293T cells upon MDP challenge. Furthermore, size exclusion chromatography revealed the formation of a complex consisting of NOD2 and NLRP1 that activates caspase-1 in response to MDP. Accordingly, unique and non-redundant functions of NOD2 and NLRP1 in formation of an MDP-responsive inflammasome that is responsible for processing and secretion of IL-1β were postulated.

Interestingly, the consequence of NOD2 mutations on the production of interleukins and the progression of Crohn's disease are controversial (reviewed in [57]). Accordingly, the effect of Crohn's disease-associated NOD2 mutations on IL-1β production differs between human and mouse macrophages. The basis for this discrepancy is not known. Consequently, Hsu et al suggest that the actual consequence of NOD2 mutations may rely on the NOD2/NLRP1 interaction [29].

In full agreement with the study of Hsu et al we found a direct interaction of NOD2 and NLRP1. Moreover our studies provide additional mechanistic insights, since we demonstrated that NOD2 selectively interacted with NLRP3 and NLRP12, but not with NLRP2, NLRP7, NLRP10, or NRLP11.

Finally, we found that a short isoform of NOD2, called NOD2-S, interacts with NLRPs, while further truncations of NOD2 did not. NOD2-S was recently described as an alternatively spliced form of NOD2 that is truncated within the second CARD domain [23]. NOD2-S was demonstrated to bind full length NOD2 and was found to inhibit NOD2 signaling in a dominant-negative fashion. Hence, NOD2-S represents a down-regulatory loop by which NOD2 is able to modulate its own activation. Whether interaction of NOD2-S with NLRP1, NLRP3 and NLRP12 also represents a down-regulatory loop for NLRP signaling remains to be investigated.

In summary, our study revealed additional layers of complexity for NLR interactions which underscores the importance of further studies to identify novel downstream effectors of NLR signaling on a genome-wide level. In this respect, the identification of a connection between NOD2 and the NLRP subfamily adds evidence to the intricate network of protein-protein interactions underlying NLR function.

## Materials and Methods

### Cloning of constructs

Coding sequences for effector domains of 11 distinct NLR proteins (NOD1, NOD2, NLRP1, NLRP2, NLRP3, etc.), three adaptor molecules (RIPK2, ASC, PYDC1), as well as five caspases (C1, C2, C4, C5, C9) were cloned in frame with the GAL4 DNA-binding (pGBKT7, Clontech) as well as the GAL4 transcription activation domain (pGADT7), respectively. Since some proteins (NOD2, NLRP1, ASC) feature more than one CARD or pyrin domain a total number of 26 distinct effector domain fusions was generated. A complete compilation of constructs is found in Table 2. All constructs were sequenced by MWG Biotech (Germany) and subsequently subjected to yeast two-hybrid analysis.

Mammalian expression vector for HA-NOD2 was a kind gift from G. Nunez. The expression vector for NLRP3 was obtained from J. Tschopp.

### Yeast two-hybrid analysis

We applied the MATCHMAKER GAL4 Two-Hybrid System 3 (CLONTECH). Two-Hybrid experiments were performed essentially as described in the manufactures instructions. Briefly, the *Saccharomyces cerevisiae* reporter strain AH109 was transformed simultaneously with certain combinations of pGBKT7-based and pGADT7-based fusion constructs using a small scale lithium acetate/single-stranded carrier DNA/polyethylene glycol (LiAc/ss-DNA/PEG) transformation protocol [58]. Transformed yeast cells were resuspended in sterile water and spotted onto SD/-Leu/-Trp dropout medium to assess transformation efficiency and onto SD/-Ade/-His/-Leu/-Trp selection medium to test for potential interactions. Plates were incubated for 4 days at 30°C. To rule out any artificial interactions all fusion constructs were pre-tested for autoactivating properties and association with either the GAL4-DNA binding or GAL4-activation domain, respectively. Co-transformation of pGBKT7-p53 and pGADT7-T served as positive interaction control, while the combination of pGBKT7-Lamc and pGADT7-T represented the negative control.

Combinations of fusion constructs positive for activation of reporter genes were confirmed by repeated small scale transformation and growth on appropriate minimal synthetic dropout media.

The strength of potential interactions was assessed by spotting transformants on SD/-His/-Leu/-Trp supplemented with increasing amounts 3-aminotriazole.

### Screening

Following initial trails, yeast transformations were conducted in an “each against all” approach, i.e. every GAL4 DNA-binding domain fusion construct was tested for association with any GAL4 transcription activation domain fusion construct, resulting in an overall number of 676 (26×26) independent transformations. The well established association between the CARDs of apoptosis protease activating factor-1 (APAF-1) and caspase-9 was considered as a paradigm for a CARD-CARD-mediated binding and served as prove of principle.

Since, all constructs were tested as both GAL4 DNA-BD as well as GAL4 AD fusions the 25 recorded associations actually corresponded to 12 unique effector domain interactions (see Table). All combinations of fusion constructs scoring positive for an interaction were validated by repeating transformations and selection on dropout medium.

### GST pull down assay

GST fusion proteins were expressed from pGEX-4T1 in *E. coli* BL21 star (Invitrogen) and affinity-purified using glutathione-Sepharose 4B beads (Amersham Pharmacia Biotech). Prey proteins were generated *in vitro* using the reticulocyte lysate TNT T7 Quick Coupled Transcription/Translation System (Promega) in the presence of [^35^S]methionine as described by the manufacturer. Purified GST fusion proteins (0.5 µg) immobilized on 15 µl of GSH-Sepharose beads were incubated with 5 µl of translation reaction containing ^35^S-labeled proteins in 500 µl binding buffer (PBS+0.1% NP-40 supplemented with 5 µg/ml Aprotinin; 5 µg/ml Leupeptin;1 mM Pefabloc SC) for 2 h at 4°C under constant agitation. Proteins on beads were washed four times in 1 ml of binding buffer. Bound proteins were eluted by boiling for 5 min in SDS sample buffer, subjected to SDS-PAGE, and analyzed by fluorography.

### In vivo co-immunoprecipitation

For immunoprecipitation, HEK293T cells (cultivated at 37°C with 5% CO2 in Dulbecco's modified Eagle's medium (Gibco-BRL) supplemented with 10% heat-inactivated fetal calf serum (Gibco-BRL) and penicillin-streptomycin (100 IU/ml and 100 mg/ml, respectively; Gibco-BRL)) were transiently transfected, using FuGene6 (Roche) according to the manufacturer's conditions, with the indicated plasmids (1 µg plasmid per 6-cm dish) and incubated for 48 h. Cells were lysed in NP-40 buffer (150 mM NaCl, 1% NP-40, 50 mM Tris-HCl, pH 7.5) containing phosphatase inhibitors (20 mM β-glycerophosphate, 5 mM NaF, 100 µM Na3VO4, and Complete protease inhibitor cocktail [Roche]). Lysates were cleared for 20 min at 14,000×g at 4°C.

Immunoprecipitation was subsequently carried out for 4 h at 4°C by adding anti-FLAG beads (M2 gel; Sigma-Aldrich) to the cell extracts. The beads were precipitated by centrifugation steps and washed five times in NP-40 buffer before sodium dodecyl sulfate loading buffer was added. Typically, about 10 to 20 times more precipitate than input was loaded into the gel. Proteins were separated by Laemmli sodium dodecyl sulfatepolyacrylamide gel electrophoresis and transferred by semidry Western transfer to a nitrocellulose membrane (Bio-Rad). Proteins were detected by incubation of the membrane subsequently with primary and secondary antibodies and by a final incubation with SuperSignal West Pico maximum sensitivity substrate (Pierce). Primary antibodies were mouse anti-FLAG M2 (1∶1000) (F3165; Sigma-Aldrich), rabbit anti-HA (1∶1,000) (sc-805; Santa Cruz Biotechnology). Secondary antibodies were horseradish peroxidase (HRP)-conjugated goat anti-mouse IgG (1∶4,000) (170-6616; Bio-Rad), HRP-conjugated goat anti-rabbit IgG (1∶4,000) (170-6515; Bio-Rad).
